# Urinary Podocyte-Associated mRNA profile in Various Stages of Diabetic Nephropathy

**DOI:** 10.1371/journal.pone.0020431

**Published:** 2011-05-31

**Authors:** Min Zheng, Lin-Li Lv, Jie Ni, Hai-Feng Ni, Qing Li, Kun-Ling Ma, Bi-Cheng Liu

**Affiliations:** Institute of Nephrology, Zhong Da Hospital, Southeast University School of Medicine, Nanjing, China; INSERM, France

## Abstract

**Background:**

Podocyte injury and subsequent excretion in urine play a crucial role in the pathogenesis and progression of diabetic nephropathy (DN). Quantification of messenger RNA (mRNA) expression in urinary sediment by real-time PCR is emerging as a noninvasive method of screening DN-associated biomarkers. We hypothesized that the urinary mRNA profile of podocyte-associated molecules may provide important clinical insight into the different stages of diabetic nephropathy.

**Methods:**

DN patients (N = 51) and healthy controls (N = 13) were enrolled in this study. DN patients were divided into a normoalbuminuria group (UAE<30 mg/g, n = 17), a microalbuminuria group (UAE 30∼300 mg/g, n = 15), and a macroalbuminuria group (UAE>300 mg/g, n = 19), according to their urinary albumin excretion (UAE). Relative mRNA abundance of synaptopodin, podocalyxin, CD2-AP, α-actin4, and podocin were quantified, and correlations between target mRNAs and clinical parameters were examined.

**Results:**

The urinary mRNA levels of all genes studied were significantly higher in the DN group compared with controls (p<0.05), and mRNA levels increased with DN progression. Urinary mRNA levels of all target genes positively correlated with both UAE and BUN. The expression of podocalyxin, CD2-AP, α-actin4, and podocin mRNA correlated with serum creatinine (r = 0.457, p = 0.001; r = 0.329, p = 0.01; r = 0.286, p = 0.021; r = 0.357, p = 0.006, respectively). Furthermore, podocalyxin mRNA was found to negatively correlate with eGFR (r = −0.349, p = 0.01).

**Conclusion:**

The urinary mRNA profiles of synaptopodin, podocalyxin, CD2-AP, α-actin4, and podocin were found to increase with the progression of DN, which suggested that quantification of podocyte-associated molecules will be useful biomarkers of DN.

## Introduction

Diabetic nephropathy (DN) is now the leading cause of end-stage renal disease (ESRD) in patients beginning renal dialysis in the United States, and this trend is extending to developing countries as well [1], [2]. The pathogenesis of DN is complex and has not yet been fully elucidated. Recent studies have shown that renal podocyte injury is pathogenically and prognostically important in DN progression. The potential mechanisms of podocyte injury include foot process effacement, hypertrophy, detachment, apoptosis, and perhaps epithelial-to-mesenchymal transition (EMT), and these mechanisms are believed to be associated with the onset and progression of DN [3]–[6]. Accumulating evidence suggests that podocyte-associated proteins or genes may correlate with proteinuria and renal function [7]–[10]. These findings bring up the interesting possibility that screening for podocyte-related molecules might be a novel strategy in monitoring the progression of DN.

Currently, renal pathological examination is the gold standard for evaluating podocytopathy in DN. However, due to the invasive nature of renal biopsy, it is impractical for physicians to closely monitor patients using this method. As a technique, real-time PCR has the benefits of excellent sensitivity, quantification, and reproducibility, and it is able to measure low-abundance genes from even one single cell [11]. In recent years, with the development of reliable RNA extraction methods from urinary sediment and real-time quantitative PCR applications, the quantification of mRNA expression in urinary sediment has become an emerging modality for studying renal pathology. Some preliminary studies suggest that the determination of urinary mRNA levels might be valuable in monitoring the progression of renal disease [12]–[15]. However, the exact clinical relevance of urinary mRNA levels remained to be determined. In this study, the expression of podocyte-associated genes in urinary sediment and their relation to disease severity were investigated in patients with DN.

## Results

### Clinical data

The primary clinical and laboratory characteristics of the study subjects are summarized in Table 1. The age of participants in the control group was lower than in the three DN groups (p<0.05), while the differences among the DN patients were not significant. The macroalbuminuria group had a significant increase in serum creatinine and BUN compared with the other three groups (p<0.001).

**Table 1:** Primary clinical and laboratory characteristics of the study subjects.

	Healthy controls(13)	Normoalbuminuria(17)	Microalbuminuria(15)	Macroalbuminuria(19)
Age (years)	50.13±10.04	67.47±10.42#	67.88±9.80#	66.06±14.39#
Sex(male/female)	9/4	13/4	12/3	14/5
Urinary albumin (mg/g(Cr))	9.06±8.68	12.00±4.89	79.00±51.35	494.06±221.71*
Serum creatinine (µmol/l)	81.43±18.94	82.95±22.67	85.24±25.73	230.42±186.20*
Blood Urea Nitrogen(mmol/l)	4.28±0.90	6.65±1.71	6.37±2.09	15.1±10.86*

#Macroalbuminuria, Microalbuminuria, and Normoalbuminuria vs. Healthy controls, p<0.05.

*Macroalbuminuria vs. Microalbuminuria, Normoalbuminuria and Healthy controls, p<0.05.

### Urinary mRNA expression levels of DN groups and healthy controls

**Figure 1:**
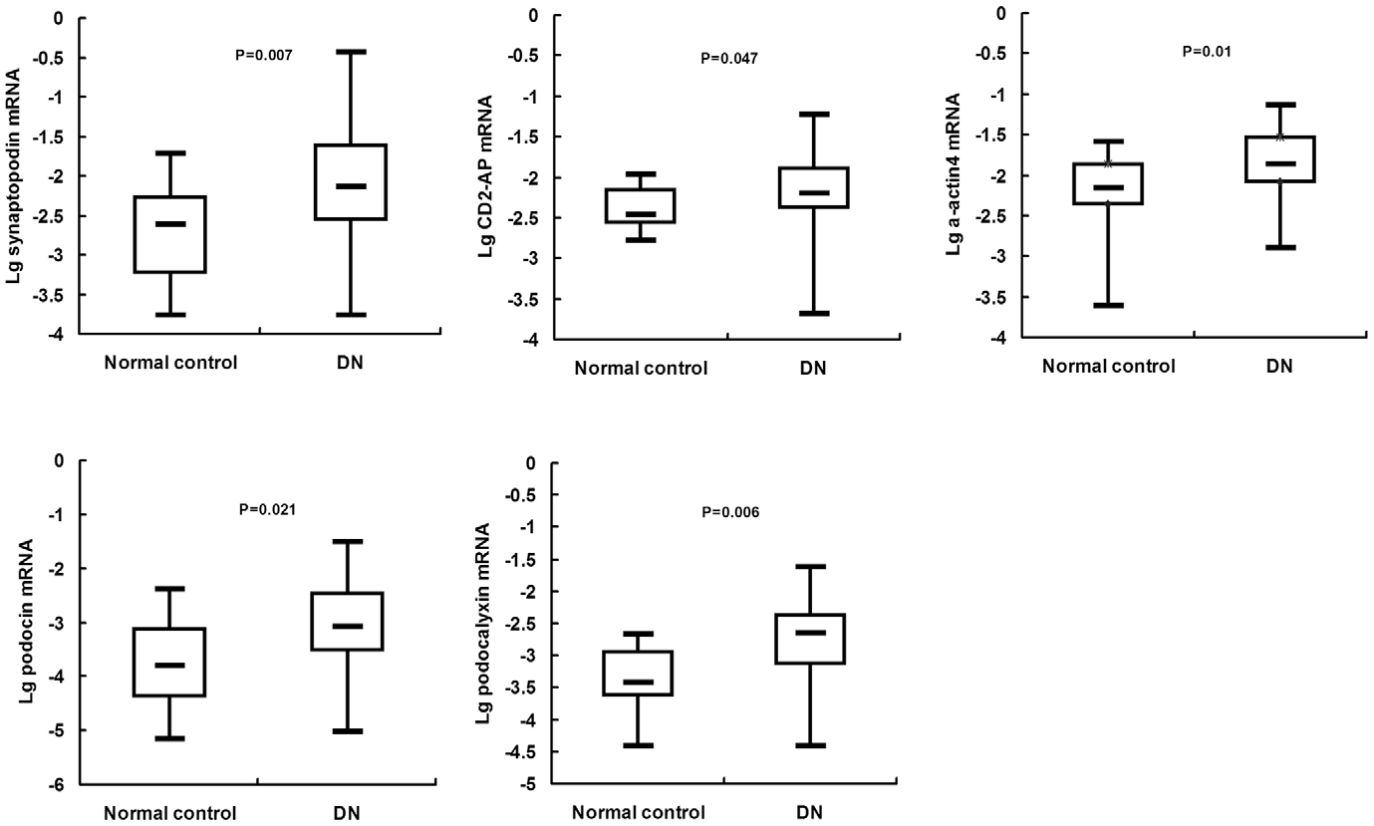
Comparison of podocyte-associated mRNA expressions in urinary between DN patients and health controls. Box plots show the minimum value, 25th, 50th (median), 75th, and the maximum values for lg-transformed ratios of mRNA copies compared with β-actin mRNA copies for synaptopodin, CD2-AP, α-actin4, podocin, and podocalyxin. Data were compared using the Mann-Whitney U test. DN  =  Diabetic nephropathy.

We first compared the levels of target gene expression between the DN groups and the healthy controls. Figure 1 summarizes the expression of the target genes synaptopodin, CD2-AP, α-actin4, podocin and podocalyxin in urinary sediment from all study subjects. The gene levels were compared by the log-transformed ratio of target gene mRNA to β-actin expression in urinary sediment cells. We found that all five target genes show significantly higher levels of expression in the experimental groups compared with the control group (p = 0.007 for synaptopodin, p = 0.047 for CD2-AP,p = 0.01 for α-actin4, p = 0.006 for podocalyxin, and p = 0.021 for podocin, respectively, by Mann-Whitney test).

### Urinary mRNA profiles and disease severity staged by albuminuria

**Figure 2:**
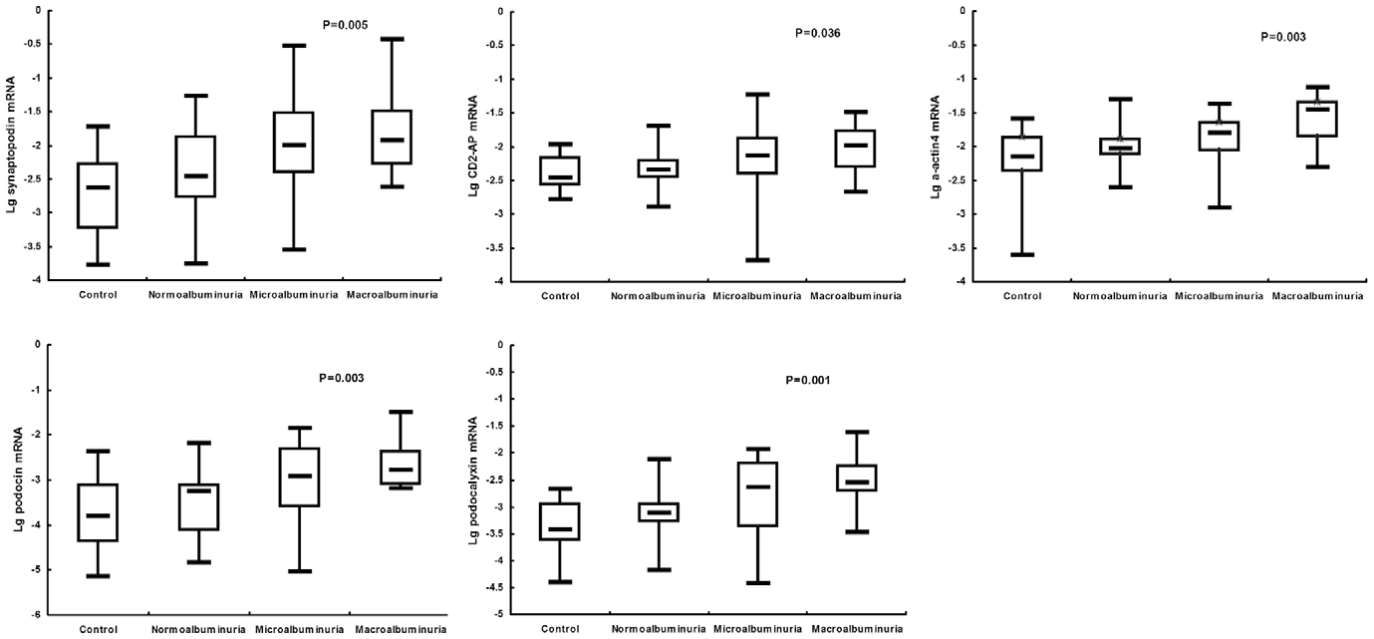
Comparison of podocyte-associated mRNA expressions in urinary in varying stages of DN and controls. Box plots show the the minimum value, 25th, 50th (median), 75th, and the maximum values for lg-transformed ratios of mRNA copies compared with β-actin mRNA copies for synaptopodin, CD2-AP, α-actin4, podocin and podocalyxin. mRNA expressions among overall four groups were compared using the Kruskal-Wallis test.

The gene expression levels in varying stages of DN (as defined by extent of albuminuria) compared with healthy controls are summarized in figure 2. The mRNA levels varied significantly among the different DN groups and healthy controls. The expression levels of all five target genes tended to increase with increasing progression of DN.

### Correlation between urinary mRNA expression and clinical parameters

**Figure 3:**
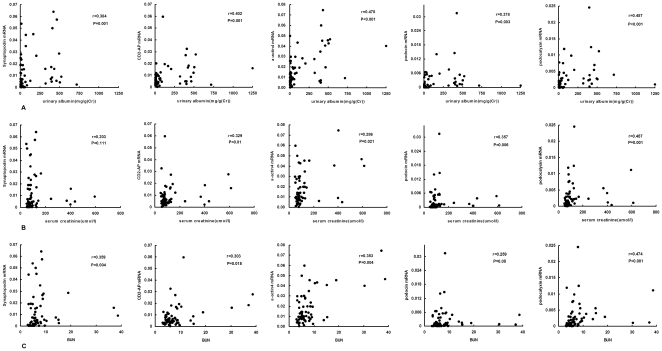
Relationships between mRNA expression of podocyte-associated molecules in urinary sediment and baseline parameters. albuminuria (A), serum creatinine (B) and BUN (C). Data were compared using the Spearman correlation coefficient.

No significant correlations between urinary mRNA expression of podocyte markers and age were found (r = 0.133, p = 0.289 for α-actin4, r = 0.018, p = 0.894 for podocin, r = −0.007, p = 0.955 for CD2-AP, r = 0.15, p = 0.279 for podocalyxin, and r = 0.12, p = 0.351 for synaptopodin using the Spearman rank-order correlation). The correlations between these markers and clinical parameters of renal function, such as urinary albumin, BUN, serum creatinine and eGFR, are summarized in figure 3. In general, the urinary mRNA levels of all target molecules were significantly correlated with urinary albumin (r = 0.478, p<0.001 for α-actin4, r = 0.378, p = 0.003 for podocin, r = 0.402, p = 0.001 for CD2-AP, r = 0.457, p<0.001 for podocalyxin, and r = 0.384, p = 0.001 for synaptopodin using the Spearman rank-order correlation). Also, a significant positive correlation was observed between BUN and urinary α-actin4 mRNA (r = 0.353, p = 0.004), CD2-AP mRNA (r = 0.303, p = 0.018), podocalyxin mRNA (r = 0.474, p<0.001) and synaptopodin mRNA levels (r = 0.359, p = 0.004). Moreover, the expression of podocalyxin, CD2-AP, α-actin4, and podocin mRNA correlated with serum creatinine levels (r = 0.457, p = 0.001; r = 0.329, p = 0.01; r = 0.286, p = 0.021; r = 0.357, p = 0.006, respectively). However, eGFR levels did not significantly relate to the mRNA levels of synaptopodin (r = −0.146, p = 0.255), CD2-AP (r = −0.209, p = 0.106), α-actin4 (r = −0.227, p = 0.069), or podocin(r = −0.202, p = 0.127), but did show a significant correlation with podocalyxin expression (r = −0.349, p = 0.01).

### ROC curve analysis of mRNA levels

**Figure 4:**
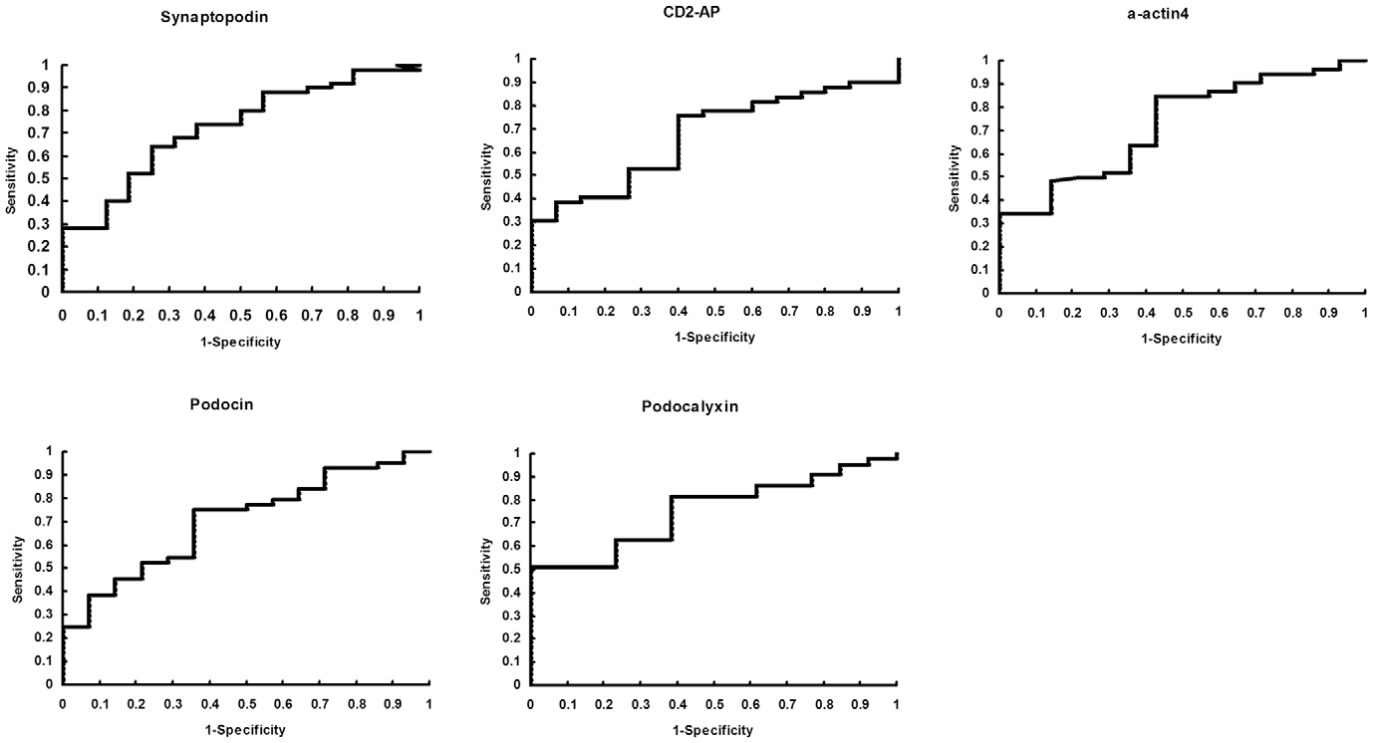
ROC-curve analysis of mRNA levels. Fraction of true positive results (sensitivity) and false positive results (1-specificity) for mRNA levels of synaptopodin, CD2-AP, α-actin4, podocin, and podocalyxin as predictors of DN. The calculated area under the curve was 0.726 for synaptopodin mRNA levels (95% confidence interval 0.589 to 0.864), 0.671 for CD2-AP mRNA levels (95% confidence interval 0.53 to 0.811), 0.725 for α-actin4 mRNA levels (95% confidence interval 0.582 to 0.867), 0.753 for podocalyxin mRNA levels (95% confidence interval 0.623 to 0.883), and 0.706 for podocin mRNA levels (95% confidence interval 0.559 to 0.853). A value of 0.5 is no better than that expected by chance (the null hypothesis), and a value of 1.0 reflects a perfect indicator.

ROC curves were calculated to assess the diagnostic power for each target gene in discriminating between DN patients and healthy controls. Figure 4 demonstrates the diagnostic performance of synaptopodin, podocalyxin, CD2-AP, α-actin4 and podocin in terms of AUCs when DN patients and controls were compared. As shown in Figure 4, all target genes were effectively able to discriminate between two groups, with an AUC above 0.5. The AUC was 0.753 (95% confidence interval, 0.623 to 0.883) for podocalyxin mRNA levels, which demonstrated the highest diagnostic value. The log-transformed threshold providing optimal sensitivity and specificity for podocalyxin mRNA was −3.24. Using the cutoff value of −3.24 derived from the data, podocalyxin mRNA levels predicted DN with a sensitivity of 81.4% and a specificity of 62.5% (p = 0.006).

## Discussion

Podocytes are highly specialized epithelial cells that cover the outer aspect of the glomerular basement membrane (GBM), playing a crucial role in the regulation of glomerular function. Both foot process effacement and a decreased number of podocytes have been documented to be associated with DN, which is a disease process that may be characterized as a podocytopathy [16]. An Akita model of type 1 DN as well as a leptin receptor-deficient db/db mouse model of type 2 DN demonstrated that diseased podocytes lose nephrin expression, become effaced, and detach from the GBM or undergo apoptosis, which all correlate with the appearance of albuminuria [17]. More recently, we demonstrated that podocytes presented with phenotypical change in the early stages of DN and that could be attenuated by irbesartan, an angiotensin II receptor antagonist [18].

A reduction in podocyte number and density has also been linked to proteinuria and progression of disease in patients with DN. A study of type 1 DN animal model demonstrated reduced podocyte number [19], and in another study, patients with renal biopsy-confirmed type 2 diabetes were confirmed to have a dramatic decrease in podocyte density [6]. A recent cross-sectional study also suggested a significant inverse correlation between proteinuria and podocyte number as well as podocyte density per glomerulus [20]. Nakamura et al. demonstrated that identification of podocytes in the urine may be a useful marker of disease activity in diabetic nephropathy [21]. However, a drawback to this modality is that it is time consuming to quantify urinary podocytes and would require an experienced cytologist. Consequently, it is important to explore alternative approaches to detect podocyte injury in order to identify novel biomarkers for DN.

Recently, efforts have focused on urinary gene expression as a potential approach for identifying biomarkers in patients with renal diseases. Li et al. demonstrated that acute renal allograft rejection could be diagnosed by quantification of perforin and granzyme B mRNA in urinary cells, and Muthukumar et al. found that mRNA measurements could improve the prediction of acute rejection outcome[22], [23]. Urinary gene expression has also been explored for monitoring therapeutic response in patients with lupus nephritis. These studies have implicated urinary mRNA detection as a means to provide us with a promising technique for diagnosing kidney disease and assessing disease activity, progression, and response to therapy [24]–[26].

In this study, we firstly determined the expression of podocyte-associated genes in the urine of patients with varying stages of DN. The results suggest that urinary synaptopodin, podocalyxin, CD2-AP, α-actin4, and podocin mRNA were significantly increased in DN patients compared with healthy controls. Increased levels of target gene expression were observed for all five podocyte markers, implying an increased excretion of podocytes into the urine of patients with DN. In a previous study, specific podocyte markers were characterized in patients with DN. Wang et al. demonstrated that urinary nephrin, podocin, synaptopodin, Wilms' tumor-1 (WT-1), and α-actin4 were increased in DN patients [27]. In the present study, we confirmed these findings and identified two more podocyte markers with high gene expression levels, providing additional candidate molecules of urinary podocyte biomarkers.

More recently, Su et al. used WT1 as a marker to evaluate podocyte damage and showed that podocyte number and density was decreased in patients with early stage of DN, and which became more dramatic as proteinuria progressed [28]. In this study, we investigated whether the expression of urinary podocyte mRNAs correlated with the progression of DN. Patients with DN were divided into three experimental groups based on their level of albuminuria. We discovered that the expression levels of all five molecules directly correlated with extent of albuminuria. Specifically, mRNA expression increased with the severity of albuminuria in experimental group, and this was consistent with previous findings describing the critical role of podocyte injury in the onset of albuminuria. The shedding of podocytes into urine might, in fact, be an important contributor in albuminuria. This result, therefore, directly supports a role for podocyte loss in the development of albuminuria [29]. To our knowledge, this is the first study to evaluate changes in urinary podocyte-associated mRNA levels in different stages of DN patients staged by albuminuria, a key indicator of progression in DN.

To further analyze the correlation between urinary mRNA levels and renal functional parameters, we identified a number of gene markers that significantly correlated with BUN and serum creatinine levels, among which α-actin4, CD2-AP, podocalyxin and synaptopodin demonstrated a positive correlation with BUN, and podocalyxin, CD2-AP, α-actin4, and podocin correlated positively with serum creatinine. eGFR, on the other hand, showed an inverse relationship with the expression of podocalyxin mRNA. It is widely accepted that podocyte injury may trigger a sequence of events through epithelial-mesenchymal transition and apoptosis or detachment, to ultimately contribute to glomerulosclerosis and decline of renal function [30]. Our current study suggests that the detection of urinary podocyte-associated mRNAs may provide valuable information for evaluating the progression of diabetic nephropathy.

In conclusion, our study demonstrates that the mRNA expression levels of synaptopodin, podocalyxin, CD2-AP, α-actin4, and podocin increase with DN progression. Quantification of podocyte-associated molecules appears to reflect the severity of albuminuria and renal damage, suggesting that these podocyte-specific genes may be used as biomarkers for DN progression.

## Materials and Methods

### Ethics Statement

All studies were approved by the Ethical Committee of Southeast University. Written informed consents were obtained from all subjects to use their urine for research purpose.

### Patient selection and clinical data

We studied 51 patients with type 2 diabetic nephropathy from Zhong Da Hospital, Southeast University. Diabetic nephropathy was diagnosed based on NKF KDOQI guidelines 2007[31]. The inclusion criteria in this study were: at least 5 years from the diagnosis of type 2 diabetes, the presence of diabetic retinopathy, elevated albumin-creatinine ratio (ACR). The exclusion criteria were: infection, signs or symptoms of other systemic disease, ACEI or ARB administration in the last 2 weeks, suspected nondiabetic kidney disease. Patients were divided into three groups based on extent of urinary albumin excretion (UAE): normoalbuminuria (UAE<30 mg/g, n = 17), microalbuminuria (UAE 30∼300 mg/g, n = 15), and macroalbuminuria (UAE>300 mg/g, n = 19). Thirteen healthy controls were from medical examination centre and the inclusion criteria were: age>40 years, non-hypertensive, non-diabetes, the absence of clinical or laboratory evidence of kidney disease. Clinical data including albuminuria, blood urea nitrogen (BUN), and serum creatinine were recorded at baseline for each of the groups. GFR was calculated by the equation proposed by Ma et al, which was considered to be more suitable for Chinese study subjects [32].

### Collection of urine samples and total RNA extraction

A whole-stream early-morning urine specimen was collected from each study participant at the first day when they were admitted to hospital. Shortly after collection, the urine was centrifuged at 3,000*g for 30 minutes at 4°C. The urinary supernatant was discarded, and the remaining cell pellet was resuspended in 1.5 ml DEPC-treated PBS and was then centrifuged at 13,000*g for 5 minutes at 4°C . The pellet was then resuspended in 1.0 ml RNAiso Plus (TAKARA, Dalian, China) and was stored at −80°C until use. Total RNA was extracted according to the manufacturer's protocol (TAKARA). All tubes and tips used for RNA extraction were 0.1% DEPC treated to inhibit the RNase and total RNA was lysised in 10–30 µl 0.1% DEPC-treated ddH2O. We confirmed the integrity of RNA by running agarose gel, which shown to be adequate for PCR. The RNA concentration and purity were confirmed using the relative absorbance ratio at 260/280 on a nanodrop 2000 (Thermo, Wilmington, USA). RNA samples with a ratio higher than 1.8 were used for RT PCR.

### Reverse transcription

For reverse transcription, 2 µg total RNA was mixed with 8 µl 5X PrimeScript™ Buffer, 2 µl PrimeScript™ RT Enzyme MixI, 2 ul Oligo dT Primer (50 µM), 2 µl Random 6 mers (100 µM), (TAKARA), the solution and was increased to a volume of 40 µl with dH_2_O. Reverse transcription was performed at 37°C for 15 minutes, followed by an inactivation reaction at 85°C for 5 seconds. The resulting cDNA was stored at −20°C until use.

### Real-time PCR

In the present study, relative abundance of synaptopodin, podocalyxin, CD2-ap, and α-actin4, podocin mRNA were quantified using the ABI Prism 7300 Sequence Detection System (Applied Biosystems, California, USA). Human β-actin was used as a reference housekeeping gene. The following oligonucleotide primer sequences were used: synaptopodin: forward 5′- CTTACGGCGGTGACATCTC, reverse 5′- GGTCCTGAGCCTCGATCC; podocalyxin: forward 5′- CTTGAGACACAGACACAGAG, reverse 5′- CCGTATGCCGCACTTATC; CD2-AP: forward 5′- AGGCTGGTGGAGTGGAAC, reverse 5′- CAGAGAAGGTATAGGTGAAGTAGG; α-actin4: 5′- GATGGTCTTGCCTTCAATG, reverse 5′- TGTTCACGATGTCCTCTG ; podocin: forward 5′- TGGCTGTGGAGGCTGAAG, reverse 5′- TGAAGGGTGTGGAGGTATCG; β-actin: forward 5′- TGGCACCCAGCACAATGAA, reverse 5′- CTAAGTCATAGTCCGCCTAGAAGCA (designed and synthesized by TAKARA). For real-time PCR, 2 µl cDNA, 10 µl SYBR Premix Ex Taq™, 0.4 µl forward primer (10 µM), 0.4 µl reverse primer (10 µM), 0.4 µl ROX Reference dye (50X; all from TAKARA) and 6.8 µl dH_2_O were mixed to make a 20 µl reaction volume. All samples were run in duplicate. The PCR technique was performed using a two-step process: 95°C for 30 s, 40 cycles at 95°C for 5 s and 60°C for 31 s. Then, dissociation curves (DC) and melting temperatures (Tm) were recorded. The results were analyzed using Sequence Detection Software version 1.4 (Applied Biosystems). The relative gene expression of each target was quantified with a standard curve method. The pre-PCR product of each gene was used as standard, and the standard curve was established with a 10-fold serial dilution of the product. The standard curve was included in all PCR runs. The equation of target gene abundance/housekeeping gene abundance was used to evaluate the level of expression of each gene. Controls consisting of ddH2O were negative in all runs.

### Statistical analysis

SPSS 13.0 was used for data analysis. All results are presented as mean±SD unless otherwise specified. Baseline data were compared by a one-way analysis of variance (ANOVA) between four groups. Since gene expression levels were highly skewed, lg transformation was used before the analysis. We used the β-actin normalized level as the dependent variable in a Kruskal-Wallis test to identify the differences between the three DN groups. The Mann–Whitney test was used for gene comparison between two groups. Correlations between gene expression and clinical parameters (urinary albumin, BUN, serum creatinine, eGFR) were calculated using the Spearman rank-order correlation. If the difference between two groups was statistically significant, the receiver-operating-characteristic (ROC) curves were established and cutoff points that yielded the highest combined sensitivity and specificity were calculated. All p-values were two tailed, and a value <0.05 was considered to be statistically significant.
